# Development and Evaluation of Self-Microemulsifying Drug Delivery System for Improving Oral Absorption of Poorly Water-Soluble Olaparib

**DOI:** 10.3390/pharmaceutics15061669

**Published:** 2023-06-07

**Authors:** Yong-Han Kim, Seong-Bo Kim, Se-Hee Choi, Thi-Thao-Linh Nguyen, Sung-Hoon Ahn, Kyung-Sun Moon, Kwan-Hyung Cho, Tae-Yong Sim, Eun-Ji Heo, Sung Tae Kim, Hyun-Suk Jung, Jun-Pil Jee, Han-Gon Choi, Dong-Jin Jang

**Affiliations:** 1 College of Pharmacy, Hanyang University, Ansan 15588, Republic of Korea; 2 Bio-Living Engineering Major, Global Leaders College, Yonsei University, Seoul 03722, Republic of Korea; 3 Department of Bio-Health Technology, College of Biomedical Science, Kangwon National University, Chuncheon 24341, Republic of Korea; 4 Department of Bio-Pharmaceutical Engineering, Kangwon National University, Chuncheon 24341, Republic of Korea; 5 College of Pharmacy, Gachon University, Incheon 21936, Republic of Korea; 6 Department of Pharmacy, College of Pharmacy, Kangwon National University, Chuncheon 24341, Republic of Korea; 7 College of Pharmacy, Inje University, Gimhae 50834, Republic of Korea; 8 Department of Artificial Intelligence, Sejong University, Seoul 05006, Republic of Korea; 9 Department of Nanoscience and Engineering, Inje University, Gimhae 50834, Republic of Korea; 10 Department of Pharmaceutical Engineering, Inje University, Gimhae 50834, Republic of Korea; 11 Department of Biochemistry, Kangwon National University, Chuncheon 24341, Republic of Korea; 12 College of Pharmacy, Chosun University, Gwangju 61452, Republic of Korea

**Keywords:** olaparib, self-microemulsifying drug delivery system, microemulsion, solubility, dissolution, oral absorption

## Abstract

The purpose of this study is to develop and evaluate a self-microemulsifying drug delivery system (SMEDDS) to improve the oral absorption of poorly water-soluble olaparib. Through the solubility test of olaparib in various oils, surfactants and co-surfactants, pharmaceutical excipients were selected. Self-emulsifying regions were identified by mixing the selected materials at various ratios, and a pseudoternary phase diagram was constructed by synthesizing these results. The various physicochemical properties of microemulsion incorporating olaparib were confirmed by investigating the morphology, particle size, zeta potential, drug content and stability. In addition, the improved dissolution and absorption of olaparib were also confirmed through a dissolution test and a pharmacokinetic study. An optimal microemulsion was generated in the formulation of Capmul^®^ MCM 10%, Labrasol^®^ 80% and PEG 400 10%. The fabricated microemulsions were well-dispersed in aqueous solutions, and it was also confirmed that they were maintained well without any problems of physical or chemical stability. The dissolution profiles of olaparib were significantly improved compared to the value of powder. Associated with the high dissolutions of olaparib, the pharmacokinetic parameters were also greatly improved. Taken together with the results mentioned above, the microemulsion could be an effective tool as a formulation for olaparib and other similar drugs.

## 1. Introduction

The Biopharmaceutics Classification System (BCS) classifies drugs into four groups based on their solubility and gastro-intestinal membrane permeability in an aqueous solution [1,2]. Among these, the drugs with low solubility but high permeability are classified as class II, while other drugs with low solubility and permeability are classified as class IV [1,2]. About 40% of currently marketed drugs and about 70% of new drug candidates under development are known as poorly water-soluble drugs, corresponding to classes II and IV [3,4,5]. In general, poorly water-soluble drugs have low oral absorption and low bioavailability due to their low solubility, which results in an increased administered dose and reduced patient compliance [5,6]. Therefore, how to improve the solubility of poorly water-soluble drugs is an important challenge in oral dosage development [7,8].

In an effort to improve the oral absorption of poorly soluble drugs, various solubilization technologies, including solid dispersion, inclusion complex, salt formation, particle size reduction, co-solvent/co-solvency and lipid-based formulations have been studied [9,10,11,12]. Of these, the lipid-based formulation is pre-dissolving drugs in lipid excipients, so it can effectively avoid the dissolution step and a potentially rate limiting dissolution step in the GI tract, and eventually has the advantage of improving bioavailability [13,14]. The liposome, solid lipid nano-carrier, nano-structured lipid carrier, emulsion and self-emulsifying drug delivery system (SMEDDS) correspond to this lipid-based formulation [15,16,17,18]. SMEDDS is an isotropic mixture of oil, surfactant and co-surfactant, and is a pharmaceutical formulation in which nano-sized emulsions are generated through spontaneous emulsification in the GI tract [19]. SMEDDS has the advantage of increased drug solubility and rapid oral absorption of the drug by the action of fine emulsion droplets, as its components, such as oil and surfactants, play the role of solubilizer [19,20]. In addition, solubilizers frequently used in SMEDDS, such as Labrasol^®^ and Tween 80, act as effective intestinal permeation enhancers and are known to be effective in increasing the oral absorption of class IV drugs [21,22]. In general, since SMEDDS is prepared in a solid dosage form, such as a gelatin capsule, the improved patient compliance is high and both manufacture and scale-up are easy, making it advantageous for commercial production [19,23]. Owing to these advantages, SMEDDS formulations have been studied to improve the in vivo bioavailability of poorly water-soluble drugs such as fenofibrate, cyclosporine and paclitaxel [24,25]. Moreover, SMEDDS has been applied to cyclosporine, ritonavir and saquinavir, and has been developed and sold as the following commercial products: Neoral^®^ (Norvatis), Norvir^®^ (AbbVie) and Fortovase^®^ (Roche) [26].

Olaparib (OLA), a poly ADP-ribose polymerase inhibitor (PARP inhibitor), has been known to have therapeutic effects for curing cancers associated with impaired DNA repair capability, especially those with deficiencies in the homologous recombination repair pathway [27]. OLA, a potent cytotoxic anticancer drug, is administrated for the treatment of patients with advanced, recurrent ovarian cancer who have mutations of breast cancer BRCA1 or breast cancer BRCA2 [28].

Despite the high therapeutic efficacies of OLA, OLA shows low oral bioavailability due to its low solubility and low permeability, which leads to increased administrated dosage and frequency [29,30]. The daily dose of Lynparza^®^ capsules is 800 mg, so the patient is obliged to endure the inconvenience of taking 16 capsules containing 50 mg of OLA every day [31,32]. This high-dose administration reduces patient compliance and triggers undesirable side effects, such as hematological toxicity, nausea, anemia, vomiting and fatigue [30,33,34]. Therefore, there is a need for a pharmaceutical formulation that can ultimately improve patient compliance and side effects by increasing the oral absorption of OLA [30].

In this research, we aimed to prepare a SMEDDS system containing OLA for improving solubility and to evaluate the obtained formulations in vitro and in vivo. Based on the results of the solubility test and the pseudoternary phase diagram, the optimal SMEDDS formulations were selected and evaluated for various physico-chemical properties, such as their morphology, particle distribution, zeta potential, dissolution profile, stability and in vivo pharmacokinetic profile. In addition, the optimal SMEDDS formulation was compared with Lynparza^®^ (a commercialized product), because our research is the first application of a microemulsion system to OLA.

## 2. Materials and Methods

**Scheme 1:**
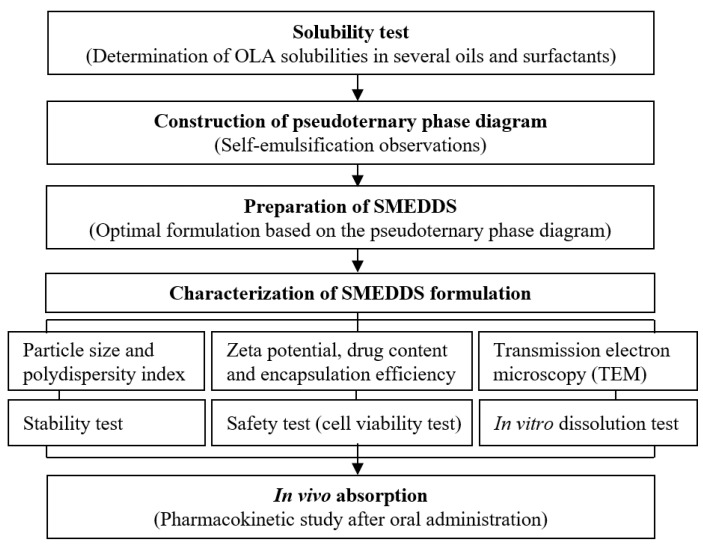
Overview of the research design in this study.

To explain the research design, a concise overview is shown in Scheme 1.

### 2.1. Materials

OLA was supplied by ScinoPharm Taiwan Ltd. (Tainan, Taiwan). Labrafil^®^ M 1944 CS, Labrafac^®^ PG, Labrasol^®^, Transcutol^®^ HP and Plurol Oleique^®^ CC 497 were supplied by Gattefosse (St. Priest, France). Cotton seed oil, Span 80, isopropyl myristate and PEG 400 were obtained from Daejung Co. Ltd. (Siheung, Republic of Korea). Oleic acid and Tween 80 were purchased from Duksan Co. Ltd. (Ansan, Republic of Korea). Mineral oil, Capmul^®^ MCM EP/NF and Kolliphor^®^ EL (known as Cremophor EL) were obtained from Samchun Chemicals Co. (Pyeongtaek, Republic of Korea), ABITEC Corp. (Columbus, OH, USA) and BASF (Ludwigshafen, Germany), respectively. MTT (3-(4,5-Dimethylthiazol-2-yl)-2,5-Diphenyltetrazolium Bromide) was purchased from Sigma-Aldrich Co. (St. Louis, MO, USA). All chemicals and solvents used were of reagent or HPLC grade.

### 2.2. Solubility Test

The solubilities of OLA were quantitatively analyzed in various aqueous solutions (distilled water, pH 1.2 and pH 6.8 solution), oils (Capmul^®^ MCM EP/NF, cotton seed oil, mineral oil, Labrafil^®^ M 1944 CS, Labrafac^®^ PG and oleic acid) and surfactants (Kolliphor^®^ EL, Tween 80, Span 80, Labrasol^®^, Transcutol^®^ HP, Plurol Oleique^®^ CC 497, isopropyl myristate and PEG 400). An excess amount of OLA was poured into each microtube containing 1.0 mL of aqueous solution, oil or surfactant. Using a Daihan RT-10 rotary mixer (Seoul, Republic of Korea), each of the blends of materials were shaken for 72 h at room temperature. Then, the tubes were centrifuged at 10,000× *g* rpm for 15 min using a Daihan CF-10 microcentrifuge (Seoul, Republic of Korea) to separate the undissolved OLA. The obtained supernatants were filtered using a 0.45 μm nylon membrane and quantitatively evaluated by a high-performance liquid chromatography system. A quantitative analysis of OLA in each filtered solution was conducted using a Hitachi Chromaster^®^ HPLC system (Tokyo, Japan) equipped with a UV detector and LB-Science Supersil 120 ODS II C_18_ column (4.6 × 250 mm, 5 μm; Dalian, China). The mobile phase comprised a mixture solution (acetonitrile: distilled water, 50:50 (*v*/*v*)). The flow rate of the mobile phase solution was 1.0 mL/min, and the column temperature was maintained at 30 °C. The detection UV wavelength was set at 200 nm.

### 2.3. Self-Emulsification and Pseudoternary Phase Diagram

Considering the solubility results for OLA in the solubility test, Capmul^®^ MCM EP/NF, Labrasol^®^ and PEG 400 were selected as the oil, surfactant and co-surfactant, respectively. To identify the self-emulsifying region of SMEDDS, the self-emulsification of a variety of compositions was meticulously observed. We added 50 μL of a mixture of Capmul^®^ MCM EP/NF, Labrasol^®^ and PEG 400 to 50 mL of distilled water, gently mixed them using a magnetic stirrer and observed their self-emulsification with naked eyes. If we obtained a nearly transparent homogenous emulsion without phase separation, it was judged that the microemulsion was successful. On the contrary, if we observed a turbid mixture or no emulsification progress, it was judged that the microemulsion was a failure. Based on the obtained self-emulsification observations, a pseudoternary phase diagram was constructed.

### 2.4. Preparation of SMEDDS

The SMEDDS formulations were selected based on the identification of a self-emulsifying region in the pseudoternary phase diagram. The formulations were prepared by mixing a certain amount of OLA, oil, surfactant and co-surfactant. A total of 100 mg of OLA was dissolved in a 3 mL mixture solution of Capmul^®^ MCM EP/NF, Labrasol^®^ and PEG 400, and mildly stirred until the mixture was clear. The obtained solutions were stored at room temperature until their physico-chemical characterizations.

### 2.5. Morphological Analysis

The morphologies of the fine emulsion droplets of the prepared SMEDDS formulation were observed using a Hitachi H-7500 transmission electron microscope (TEM) system (Tokyo, Japan). After the dilution of the sample solution with distilled water, the samples were deposited on the carbon-coated grid and dried at room temperature. Then, the dried samples were stained with 2% (*w*/*v*) phosphotungstic acid solution for 2 min prior to observation.

### 2.6. Droplet Size and Zeta Potential Analysis

The droplet size distribution and zeta potential of the prepared microemulsions were evaluated by an Otsuka ELSZ-100 particle size analyzer (Tokyo, Japan). A total of 5 μL of the prepared microemulsion was added to 50 mL of distilled water and the diluted sample was analyzed at room temperature (*n* = 3). The particle size values and distribution were calculated from the intensity-weighted distribution and polydispersity index (PDI), respectively.

### 2.7. Stability Test

To evaluate the storage stability of the SMEDDS formulation, the samples were tightly sealed in 5 mL glass vials. According to the ICH guidelines (Q1), the samples were stored for 3 months under long-term storage conditions (25 ± 2 °C, 60 ± 5% RH) and accelerated storage conditions (40 ± 2 °C, 75 ± 5% RH). A storage stability test was conducted on the physical application, emulsification, droplet distribution, zeta potential and OLA contents at 1 and 3 months after the initial time. Each sample was evaluated three times and the data were processed.

### 2.8. In Vitro Dissolution Test

An in vitro dissolution study of OLA was conducted using a Kukje KDT-600 dissolution tester (Goyang, Republic of Korea) under the USP apparatus II (paddle method). The dissolution media were HCl buffer (pH 1.2), phosphate buffer (pH 6.8) and distilled water. The paddles were rotated at 100 rpm and the temperature was 37 °C. The SMEDDS system (equivalent to OLA 10 mg) was filled into capsules (hard gelatin) prior to the dissolution test and compared with the OLA powder. During the dissolution test, each dissolution medium (3 mL) was taken at predetermined times (5, 10, 15, 30, 45, 60, 90 and 120 min) and filtered through a 0.45 μm syringe filter. The obtained aliquots were quantitatively analyzed using the HPLC system as described in Section 2.2. After taking dissolution samples, an equal volume of the dissolution medium was replaced at each time point and all the experiments were conducted three times. The statistical significances between the results of the OLA powder and SMEDDS formulation were confirmed by the student’s t-test method using Jandel scientific Sigmaplot version 12.0 (San Rafael, CA, USA).

### 2.9. In Vivo Pharmacokinetic Study

To confirm the improvement of the oral absorption of OLA contained in the SMEDDS formulation, a rat pharmacokinetic study of the OLA powder, SMEDDS formulation and Lynparza^®^ tablet was conducted. The rats used in this experiment were fasted overnight, but water was freely consumed. Blood samples were taken from the jugular vein using a 1.0 mL syringe treated with heparin at predetermined times (0, 30, 60, 120, 240, 480 and 1440 min) after the oral administration of each formulation. The blood samples were centrifuged at 7000× *g* rpm and 4 °C for 10 min, and the obtained plasma was stored at −80 °C until the quantitative analysis.

A total of 50 μL of the plasma samples was added to 150 μL of an internal standard solution (carbamazepine 200 ng/mL in acetonitrile), then mixed on a vortex mixer for 3 min. After centrifugation for 10 min at 13,000× *g* rpm at 4 °C, the supernatants were transferred into new LC vials and analyzed by injecting 5 μL of the supernatants into an LC-MS/MS system. The autosampler was set at 10 °C during the analysis. The chromatographic separation of OLA was conducted on a Gemini-NX C18 column (50 × 2.0 mm, 3 μm particle size, 110 Å; Phenomenex Inc., Torrance, CA, USA) using an isocratic elution condition of acetonitrile and 10 mM of ammonium formate buffer (80:20, *v*/*v*) at a flow rate of 0.3 mL/min and a column oven temperature of 40 °C, using the Agilent 1200 series HPLC system (Agilent Technologies, Santa Clara, CA, USA). The method of mass spectrometry known as multiple reaction monitoring (MRM) was utilized for the analytical procedure, and the following parameters were used: an ion-spray voltage of + 5500 V at 550 °C; a decluttering potential of 111 V; an entrance potential of 11 V; a collision energy of 37 V and a mass transition m/z of 435.1 > 281.3 for OLA.

The following pharmacokinetic parameters were calculated using the WinNonlin program version 5.0 (Princeton, NJ, USA): the time to reach the maximum plasma concentration (T_max_); the maximum plasma concentration (C_max_); the area under the plasma concentration-time curve to the last time point or infinity time (AUC_last_ or AUC_inf_,) and the half-life (T_1/2_). Additionally, the relative oral bioavailability (BA) was calculated by dividing the AUC for the SMEDDS formulation or the Lynparza^®^ tablet by that of OLA. All data were expressed as means ± S.D. The student’s *t*-test was conducted to determine statistically significant differences (*p* < 0.05) among the tested groups.

### 2.10. In Vitro Cytotoxicity Assay

The cell viability values of the formulated SMEDDS system were evaluated by an MTT assay using the CT26 cells (human colon carcinoma). RPMI 1640 media supplemented with FBS (10%), penicillin (100 units/mL) and streptomycin (100 mg/mL) were used as the culture media for the CT26 cell line at 37 °C and 5% CO_2_. The cultured cell lines were placed at a density of about 1 × 10^4^ cells/well in well microplates and allowed to adhere onto the bottom of the wells. The cell lines attached to the wells were washed with phosphate buffered saline (PBS) and incubated for 24 h with various concentrations of SMEDDS solution without OLA. The treated cells were washed with PBS, treated with the MTT solution (5 mg/mL in PBS) and incubated again at 37 °C for 6 h. The cell viability (%) in the obtained solution was evaluated at 570 nm using a UV spectrophotometer and compared with untreated control samples.

## 3. Results and Discussion

### 3.1. Solubility Test of OLA

Solubility tests of OLA in various aqueous solutions, oils and surfactants were performed (Table 1). The aqueous solubility of OLA was 0.073 ± 0.25 mg/mL in distilled water, 0.063 ± 0.57 mg/mL at a pH of 1.2 and 0.059 ± 0.42 mg/mL at a pH of 6.8, consistently very low regardless of pH. These results show that, even after the oral administration of OLA with a daily dose of 800 mg, most of the OLA is not dissolved in the GI tract and is precipitated in an insoluble state, which may lead to very low oral absorption. In general, it is known that the lower the solubility of a drug, the lower the bioavailability, which increases the administered dose, as well as side effects [30].

Improving the drug solubility using an appropriate solubilizer may increase the oral absorption, thereby decreasing the administrated dose. As a result, this study conducted a screening test of excipients frequently used in the pharmaceutical field to search for substances that can improve the solubility of OLA. Among various oils, the solubility of Capmul^®^ MCM EP/NF was about 574 times that of distilled water (41.68 ± 0.02 mg/mL vs. 0.07 ± 0.25 mg/mL). In the case of surfactants, the solubilities of PEG 400 and Labrasol^®^ were 47.66 ± 0.38 mg/mL and 36.60 ± 0.07 mg/mL, respectively, which were improvements of about 657.3 times and 504.8 times, respectively, compared to distilled water. Through the excipient solubility screening test described above, Capmul^®^ MCM EP/NF, PEG 400 and Labrasol^®^ were selected as the oil, surfactant and co-surfactant to be used in the production of OLA SMEDDS.

### 3.2. Construction of a Pseudoternary Phase Diagram

**Figure 1:**
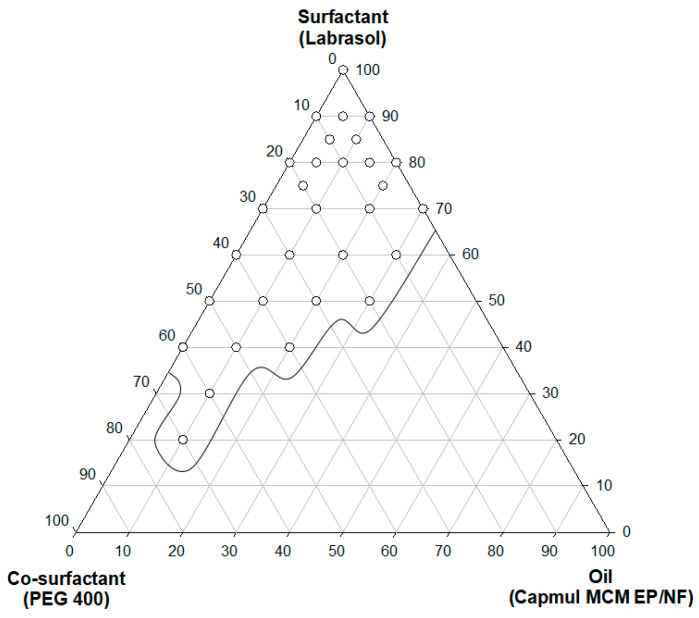
Pseudoternary phase diagram of oil, surfactant and co-surfactant. (Capmul^®^ MCM EP/NF, Labrasol^®^ and PEG 400, respectively).

A self-emulsification assessment was performed using the selected oil (Capmul^®^ MCM EP/NF) and surfactant (PEG 400 or Labrasol^®^). Various SMEDDS mixtures were prepared by diversifying the ratio of the selected substances to proceed with various evaluations. By constructing a pseudoternary phase diagram based on the obtained results, self-emulsifying regions could be identified. The region shown on Figure 1 represents a stable self-emulsifying region, either clear or slightly bluish. When the oil ratio was 5–30%, an excellent microemulsion was observed.

It was confirmed that the higher the oil ratio, the larger the droplet size of the emulsion. When checking the pseudoternary phase diagram, the higher the ratio of Labrasol^®^ or PEG 400 that was used as surfactant, rather than Capmul^®^ MCM EP/NF, the better the emulsions that were generated. This phenomenon is mentioned in several studies, revealing that as the proportion of surfactant and co-surfactant increases, the interface of the emulsion becomes more stabilized and condensed [35]. Based on the pseudoternary phase diagram, Capmul^®^ MCM EP/NF 10%, Labrasol^®^ 80% and PEG 400 10% were selected as the optimal SMEDDS production ratio for OLA. Considering the solubility of OLA in each substance, a SMEDDS formulation containing 100 mg of OLA in a mixture of Capmul^®^ MCM EP/NF 0.3 mL, Labrasol^®^ 2.4 mL and PEG 400 0.3 mL was obtained. Using this resultant formulation, several characterizations were investigated.

### 3.3. Characterization of SMEDDS

**Figure 2:**
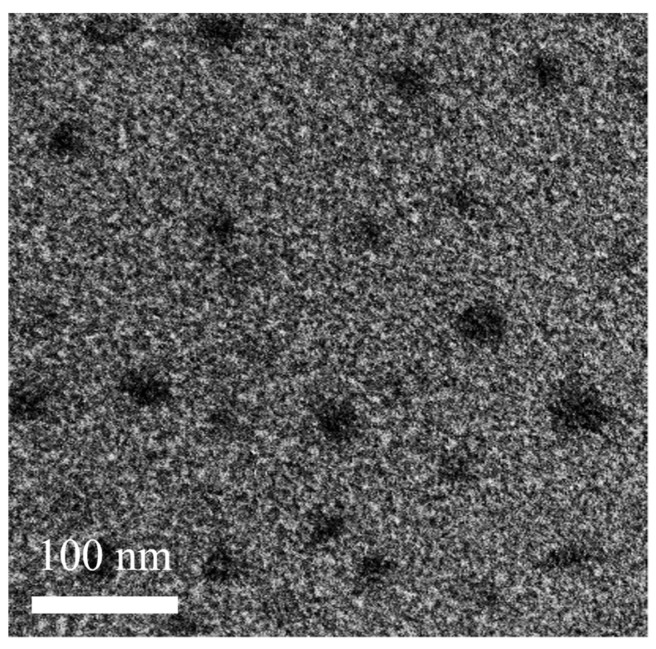
Transmission electron microscopy image of OLA SMEDDS.

The morphology of the prepared SMEDDS was observed using TEM. Figure 2 shows that the microemulsion droplets are well dispersed. The mean droplet size was 141.1 ± 0.2 nm, and the polydispersity index was very small, 0.19. In general, having a PDI value of 0.2 or less in a dispersed system means that the particle size distribution is quite narrow [36]. The zeta potential was measured at −15.8 ± 1.1 mV, and it has been reported that nano-sized dispersed systems with a zeta potential lower than 10 mV can maintain physical stability and improve drug efficacy by improving in vivo absorption [37]. Judging by the obtained test results and the results of various other studies, it was expected that the manufactured OLA SMEDDS system could show high physical stability and oral absorption of OLA because it had the appropriate physical properties.

### 3.4. Stability Study of SMEDDS

To evaluate the stability of the obtained SMEDDS formulation, the physico-chemical properties were checked while storing the fabricated samples under the long-term stability test conditions (25 ± 2 °C, 60 ± 5% RH) and accelerated stability test conditions (40 ± 2 °C, 75 ± 5% RH) (Table 2). During the storage period, the formulation’s physical stability was confirmed as it maintained its clear liquid state without drug precipitation and phase separation. In addition, when the SMEDDS formulation was dispersed in distilled water, the self-emulsification was also confirmed to have maintained its initial state. For 3 months, the droplet size increased from 141. 1 ± 0.2 nm to 166.1 ± 2.7 nm, and the zeta potential slightly increased from −13.5 ± 0.8 mV to −18.8 ± 0.4 mV, but the nano-sized dispersion still remained stable. Lipid- or oil-based particulate systems can maintain their physical stability thanks to the electrostatic effects of the surface charge and steric stabilization from the PEG chain [38]. The physical stability of the microemulsions manufactured in this study could be maintained by a negative charge with an absolute value of more than 10 mV and steric stabilization generated by the addition of PEG. The drug contents were 101.0 ± 0.2% and 98.5 ± 0.2% in long term storage and accelerated storage, respectively, which is not significantly different from the initial value (101.1 ± 0.5%). Because the SMEDDS system does not contain water in the pharmaceutical formulation, it is generally known to have a relatively high stability compared to other liquid-type dosage formulations [23]. Taken together, these results show that the self-emulsification behavior was maintained and the physical properties of the SMEDDS formulation did not change significantly.

### 3.5. In Vitro Dissolution Study

**Figure 3:**
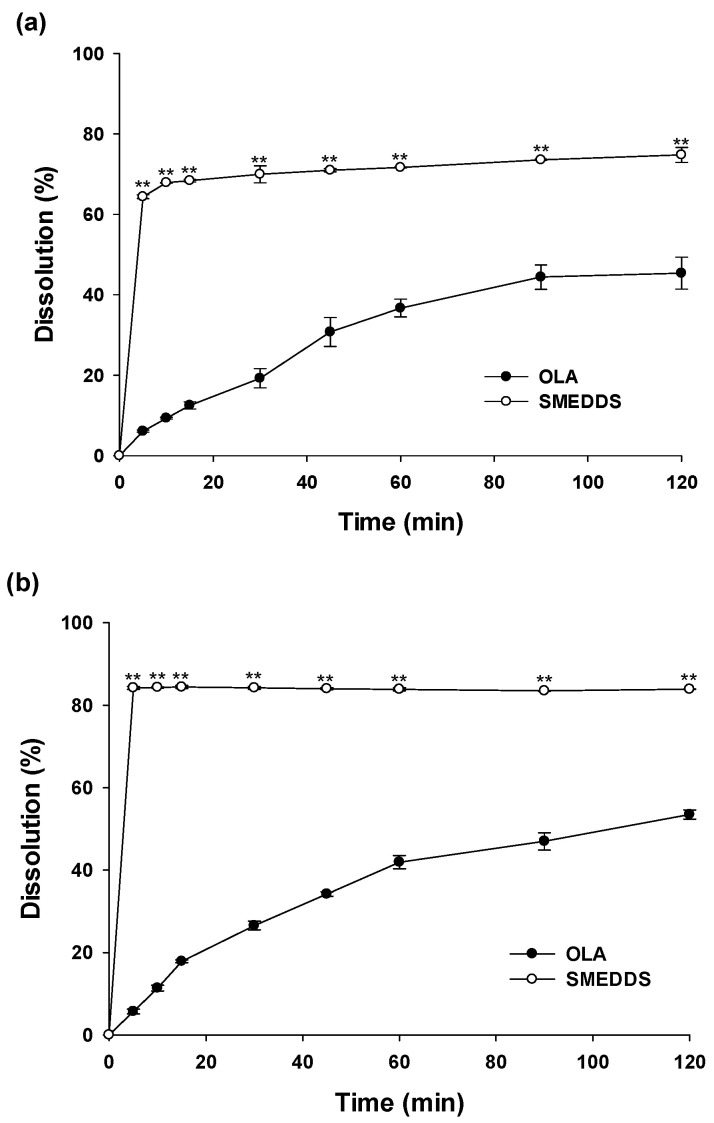

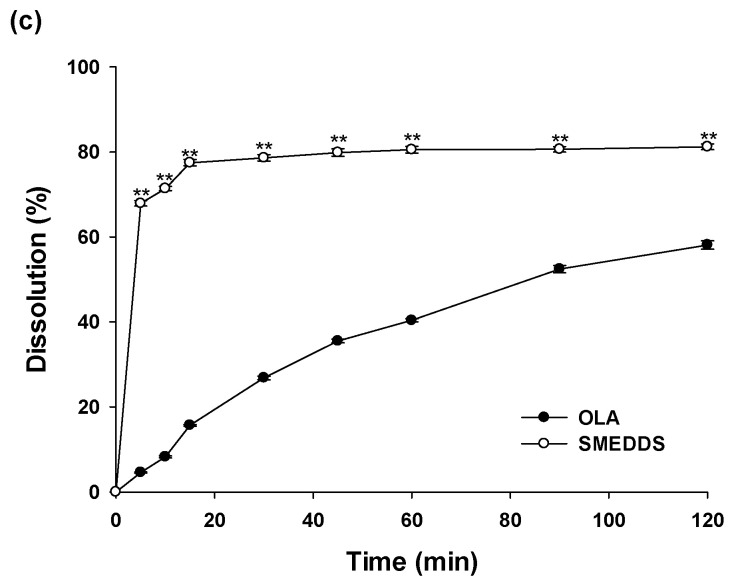
In vitro dissolution study of SMEDDS and OLA powder. Dissolution profile in (**a**) HCl buffer (pH 1.2); (**b**) phosphate buffer (pH 6.8); (**c**) distilled water; ** *p* < 0.01 compared with OLA.

The in vitro dissolution profiles of OLA from the SMEDDS formulation were monitored and compared with the OLA powder under different pH conditions (pH 1.2, pH 6.8 and distilled water) for 2 h (Figure 3). In the pH 1.2 solution, the dissolutions of the OLA powder were only 6.09 ± 0.27% within 5 min and 45.39 ± 3.97% at 120 min. On the other hand, that of OLA in the SMEDDS formulation was 64.38 ± 0.48% within 5 min, showing a very rapid dissolution profile, followed by a final dissolution of 74.81 ± 1.87% at 120 min. Similar results to those at a pH of 1.2 were also obtained at a pH of 6.8 and in distilled water. At a pH of 6.8 and in distilled water, the dissolution values of OLA in the SMEDDS system were 84.20 ± 0.31% and 67.93 ± 0.64%, respectively, for the initial 5 minutes. In contrast, those of the OLA powders were very low at 5.70 ± 0.59% and 4.58 ± 0.14%, respectively. According to the solubility results, the solubility of OLA is independent of the pH conditions. Therefore, it is estimated that the dissolution profiles at a pH of 1.2, a pH of 6.8 and in distilled water are all similar. The dissolutions of OLA contained in SMEDDS at the initial 5 min had improved 10.57 times (pH 1.2), 14.78 times (pH 6.8) and 14.83 times (distilled water) compared to the OLA powder, and the final dissolution rates (at 120 min) had improved 1.65 times (pH 1.2), 1.57 times (pH 6.8) and 1.52 times (distilled water). The improved dissolution rates of the drugs contained in SMEDDS formulations has been reported in several documents. These results showed that the improved dissolutions of the SMEDDS formulation containing OLA were because of the increased specific surface area of nano-sized microemulsion droplets and the solubilization effects of optimal oils and surfactants. As such, the SMEDDS formulation showed an improved dissolution profile compared to the OLA powder, which would lead to the enhanced oral adsorption of OLA.

### 3.6. In Vivo Pharmacokinetic Study and In Vitro Cytotoxicity

**Figure 4:**
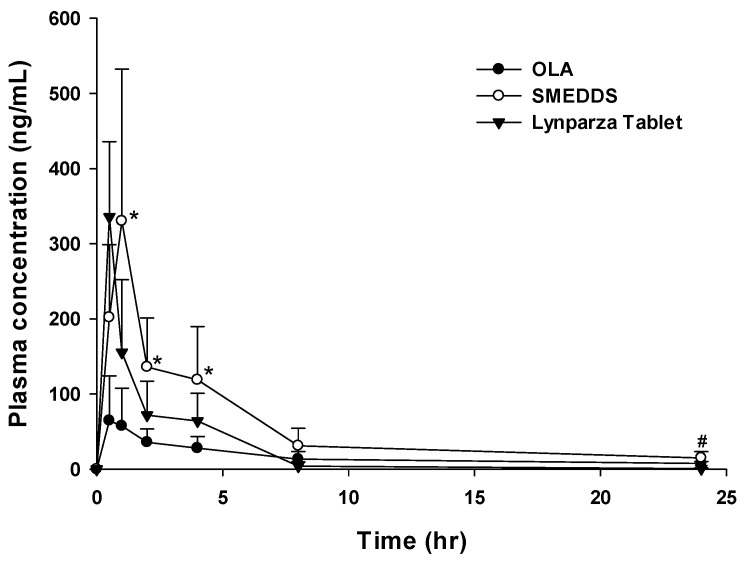
Plasma concentration-time profiles in rats after oral administration of OLA powder, SMEDDS and Lynparza^®^ tablet; * *p* < 0.05 compared with OLA; ^#^ *p* < 0.05 compared with Lynparza^®^ tablet.

The in vivo pharmacokinetic behavior of OLA with SMEDDS, a marketed product (Lynparza^®^ tablet) and OLA powder was investigated in rats. The mean plasma concentration profiles of OLA were plotted as a function of time, as presented in Figure 4. The mean pharmacokinetic parameters of OLA absorption are summarized in Table 3. The C_max_ and AUC_last_ of OLA powder were 83.1 ± 49.6 ng/mL and 384.1 ± 136.2 ng·h/mL, respectively. The C_max_ and AUC_last_ of the Lynparza^®^ tablet were 335.7 ± 100.2 ng/mL and 518.2 ± 154.9 ng·h/mL, respectively. On the other hand, the C_max_ and AUC_last_ following SMEDDS administration were 351.2 ± 173.3 ng/mL and 1242.7 ± 420.3 ng·h/mL, a significant increase of 4.2 and 3.2, respectively, compared to the OLA powder. When compared with the Lynparza^®^ tablet, the C_max_ was similar, but the AUC_last_ was significantly increased, by 2.4. The AUC_inf_ of the OLA powder and the Lynparza^®^ tablet were 634.0 ± 437.2 ng·h/mL and 522.0 ± 155.9 ng·h/mL, respectively. The AUC_inf_ of SMEDDS was 1427.3 ± 460.3 ng·h/mL, which was an increase of 2.25 compared to the OLA powder (634.0 ± 437.2 ng·h/mL) and of 2.73 compared to the Lynparza^®^ tablet (522.0 ± 155.9 ng·h/mL), respectively. The T_max_ of SMEDDS was slightly delayed compared to the Lynparza^®^ tablet, but the T_1/2_ showed there was no significant difference among the formulations. To improve the low oral bioavailability of OLA, several nano-sized formulations (lipo-sphere nanoparticles (LP-NP) by melt dispersion and nano-suspensions (NSP) by solvent evaporation (SE) and wet milling (WM) techniques) have been studied. These dispersion systems are known to be very effective options for improving the oral absorption of poorly water-soluble drugs. The oral AUC of LP-NP, NSP-SE and NSP-WM in rats significantly increased by 1.5, 1.9 and 1.4, respectively [30]. In this study, the AUC of the OLA SMEDDS increased by 3.2, proving that SMEDDS can be used as an effective formulation for improving the oral absorption of OLA (Table 3).

In most cases, the toxicity of anticancer drugs increases in proportion to the administered dose [39]. In particular, it is known that the high-dose administration of OLA can cause side effects such as gastrointestinal toxicities, fatigue and anemia, and therefore, dose reduction through formulation improvement can reduce toxicity [40]. As a result, when analyzing the results of the pharmacokinetic study conducted in this study comprehensively, the SMEDDS formulation is expected to significantly reduce the dosage and toxicity compared to the marketed formulation.

Additionally, Capmul^®^ MCM EP/NF (oil) [41,42], Labrasol^®^ (surfactant) [43,44] and PEG 400 (co-surfactant) [45,46] are widely employed as biocompatible additives and were incorporated into the pharmaceutical formulations. The use of biocompatible materials in drug development is important because it could reduce the risk of acute toxicity and chronic toxicity. In this study, the SMEDDS formulation containing the above-mentioned safe substances showed almost no cytotoxicity in a safety test using the MTT assay. We confirmed that the cell viability (%) of the pretreated media with a fabricated formulation was over 97% (<3% cell death at all concentrations). This result indicates that the prepared microemulsion has almost no cytotoxic effects on the CT26 cell line. Furthermore, no acute toxic-induced death was observed up to or over 24 h after oral administration in rats. 

## 4. Conclusions

The optimized SMEDDS containing OLA was developed using Capmul^®^ MCM EP/NF, Labrasol^®^ and PEG 400. This formulation system formed a stable microemulsion system for at least 3 months under long-term storage conditions (25 ± 2 °C, 60 ± 5% RH) and accelerated storage conditions (40 ± 2 °C, 75 ± 5% RH). In addition, it showed higher dissolution patterns than OLA powder and even the commercial formulation. The in vivo pharmacokinetic results also showed the improvement of OLA oral absorption. In conclusion, this system suggests the potential use of SMEDDS for the oral administration of OLA or other hydrophobic drugs.

## Figures and Tables

**Table 1:** Solubility of OLA in various aqueous solutions, oils, surfactants and co-surfactants (mean ± S.D.; *n* = 3).

Vehicle	Solubility (mg/mL)
Aqueous solution	
Distilled water	0.07 ± 0.25
pH 1.2	0.06 ± 0.57
pH 6.8	0.06 ± 0.42
Oils	
Capmul^®^ MCM EP/NF	41.68 ± 0.02
Cotton Seed Oil	0.06 ± 0.20
Labrafil^®^ M 1944 CS	0.95 ± 0.71
Labrafac^®^ PG	0.13 ± 0.10
Oleic acid	3.83 ± 0.22
Surfactants and co-surfactants	
Kolliphor^®^ EL	7.83 ± 6.06
Tween 80	8.82 ± 0.28
Span 80	4.82 ± 3.52
Labrasol^®^	36.60 ± 0.07
Transcutol^®^ HP	19.69 ± 0.14
Plurol Oleique^®^ CC 497	4.99 ± 0.09
Isopropyl Myristate	0.04 ± 0.02
PEG 400	47.66 ± 0.38

**Table 2:** Stability study of SMEDDS at 25 °C, 60% RH and 40 °C, 75% RH (Mean ± S.D.; *n* = 3).

Time (month)	Droplet Size (nm)	Zeta Potential (mV)	Drug Content (%)
(a) 25 °C, 60% RH			
0	141.1 ± 0.2	−15.8 ± 1.1	101.1 ± 0.5
1	142.7 ± 0.4	−18.8 ± 0.4	101.1 ± 0.2
3	164.1 ± 0.3	−16.3 ± 0.7	101.0 ± 0.2
(b) 40 °C, 75% RH			
0	141.1 ± 0.2	−15.8 ± 1.1	101.1 ± 0.5
1	143.0 ± 0.7	−13.5 ± 0.8	99.0 ± 0.1
3	166.1 ± 2.7	−16.0 ± 0.7	98.5 ± 0.2

**Table 3:** Pharmacokinetic parameters of SMEDDS and OLA at an equivalent dose of 10 mg/kg in rats (mean ± S.D.; *n* = 5); * *p* < 0.05 and ** *p* < 0.01 compared with OLA; ^#^ *p* < 0.05 compared with Lynparza^®^ tablet.

PK Parameters	OLA	Lynparza^®^ Tablet	SMEDDS
T_max_ (h)	0.8 ± 0.3	0.5 ± 0.0	0.9 ± 0.2 ^#^
C_max_ (ng/mL)	83.1 ± 49.6	335.7 ± 100.2	351.2 ± 173.3 *
T_1/2_ (h)	9.4 ± 0.1	4.0 ± 0.9	7.3 ± 3.2
AUC_last_ (ng·h/mL)	384.1 ± 136.2	518.2 ± 154.9	1242.7 ± 420.3 **^,#^
AUC_inf_ (ng·h/mL)	634.0 ± 437.2	522.0 ± 155.9	1427.3 ± 460.3 *^,#^

## Data Availability

Not applicable.
